# Enhancing nutrition knowledge and dietary diversity among rural pregnant women in Malawi: a randomized controlled trial

**DOI:** 10.1186/s12884-021-04117-5

**Published:** 2021-09-22

**Authors:** Lillian Ziyenda Katenga-Kaunda, Penjani Rhoda Kamudoni, Gerd Holmboe-Ottesen, Heidi E. Fjeld, Ibrahimu Mdala, Zumin Shi, Per Ole Iversen

**Affiliations:** 1 Malawi Government, Health and Social Services, Mzuzu City Council, Mzuzu, Malawi; 2 Department of Nutrition, University of Oslo, Oslo, Norway; 3 Department of Community Medicine and Global Health, University of Oslo, Oslo, Norway; 4 QU Health, Qatar University, Doha, Qatar; 5 Department of Haematology, Oslo University Hospital, Oslo, Norway; 6 Division of Human Nutrition, Stellenbosch University, Tygerberg, South Africa

**Keywords:** Diet, Education, Foods, Pregnancy

## Abstract

**Background:**

In many sub-Saharan African countries, such as Malawi, antenatal care (ANC) services do not deliver sufficient nutrition awareness to improve adequate dietary intake in pregnancy. We therefore compared the effects of supplementary nutrition education and dietary counselling with routine ANC service on nutrition knowledge and dietary intakes among Malawian pregnant women.

**Methods:**

We used data from a two-armed cluster randomised controlled trial (RCT) of which the intervention group received supplementary nutrition education, dietary counselling and routine ANC services whereas the controls received only routine ANC services. The RCT was conducted in 10 control and 10 intervention villages in Mangochi, Southern Malawi and included pregnant women between their 9^th^ and 16^th^ gestational weeks. We examined the changes in nutrition knowledge and dietary diversity from enrolment (baseline) to study end-point of the RCT (two weeks before expected delivery). We used three linear multilevel regression models with random effects at village level (cluster) to examine the associations between indicators of nutrition knowledge and diet consumption adjusted for selected explanatory variables.

**Results:**

Among 257 pregnant women enrolled to the RCT, 195 (76%) were available for the current study. The supplementary nutrition education and counselling led to significant improvements in nutrition knowledge, dietary diversity and nutrition behaviour in the intervention group compared with controls. Most women from both study groups had a moderate consumption of diversified foods at study end-point. A significant positive association between nutrition knowledge and consumption of a diversified diet was only observed in the intervention group.

**Conclusions:**

Nutrition knowledge and dietary diversity improved in both study groups, but higher in the intervention group. Increased nutrition knowledge was associated with improved dietary diversity only in the intervention women, who also improved their nutrition perceptions and behaviour. Antenatal nutrition education needs strengthening to improve dietary intakes in pregnancy in this low resource-setting.

**Trial registration:**

Clinical trials.gov ID: NCT03136393 (registered on 02/05/2017).

## Background

Antenatal care (ANC) is the regular medical and nursing care for pregnant women, including the provision of nutrition education and counselling services. ANC is often decentralised and hence a locally accessible platform for the delivery of maternal nutrition interventions in low-resource settings, such as sub-Saharan Africa [1]. In Malawi in 2016, 80% of pregnant women reported that their main source of nutrition information was ANC services [2]. World Health Organisation (WHO)-revised guidelines for undernourished populations emphasise the relevance of nutrition education and counselling services for ANC, aiming to increase daily energy and protein intake to reduce the risk of low birth weight (LBW, birth weight < 2,500 g) infants [3]. This is particularly relevant for Malawi, where maternal malnutrition accounts for more than half of the LBW babies [4].

Nutrition education is defined as any combination of educational strategies, accompanied by environmental support, designed to facilitate voluntary adoption of food choices and nutrition-related behaviours beneficial to health and well-being [5]. Nutrition education plays a pivotal role in nutrition behaviour change efforts, as it can enhance participants’ attainment of nutrition- and food literacy. Nutrition literacy refers to the set of abilities needed to understand and interpret information about food and their nutrients, while food literacy encompasses nutrition literacy as well as the ability to apply that information in making appropriate decisions [6, 7]. Knowing the beneficial effects of good nutrition and having skills to prepare the food can motivate the pregnant women to develop intentions to improve their dietary habits [5].

Food literacy in the Malawian context includes the ability to understand the benefits of good nutrition by applying the Six Food Group (SFG) which is a national recommended guideline for measuring dietary quality [8]. The SFG is an example of food group diversity that can be translated to a score to assess dietary quality and consists of staple (grains), vegetables, animal/fish food, legumes, fruits, and fats [9]. The Dietary Diversity Score (DDS) is another tool often used internationally for assessing dietary quality in micronutrients [10]. It includes 10 food groups; being grains, nuts/seeds, dairy, meat/poultry/fish, eggs, dark green leafy vegetables, other vegetables, vitamin A rich fruits, other fruits, and legumes/pulses.

Nutrition education/counselling is only one of 15 topics that constitute the educational component of the Malawian ANC services. These competing topics to be addressed during the eight antenatal visits recommended by WHO may lead to situations where pregnant women are unable to access quality nutrition education and counselling through the ANC services [11–15]. In line with this, studies have found that care providers often have insufficient time to facilitate group- and individual education sessions, attributing this to high client/provider ratios during the ANC visits [16–18]. Furthermore, attendance to antenatal visits remains low among women in poor Malawian communities, which implies that many pregnant women miss out on substantial education components offered through ANC services [2]. These factors raise uncertainties about the effect that nutrition education counselling through routine ANC have on developing the required levels of nutrition- and food literacy.

There are limited published research on the contributions of ANC to food literacy among Malawian pregnant women, and there is a need for evidence on the effectiveness of ANC in nutrition education in order to inform the development of appropriate interventions for this vulnerable population. We previously conducted a cluster randomised controlled trial (RCT) among pregnant women in rural Malawi with the intention to increase birth weight of their babies, nutritional knowledge and to improve their dietary habits. Here we used data from this RCT to describe the differences in nutrition knowledge and dietary habits between women who attended only routine ANC (control group) and women who accessed a supplementary community-based nutrition education and counselling (intervention group). Specifically, we wanted to (i) describe and compare changes in nutrition knowledge, perception and dietary habits at study baseline and study end-point between the intervention and control groups; and (ii) to evaluate the effects of nutrition knowledge on dietary diversification among these two study groups.

## Methods

### Study-setting

**Fig. 1:**
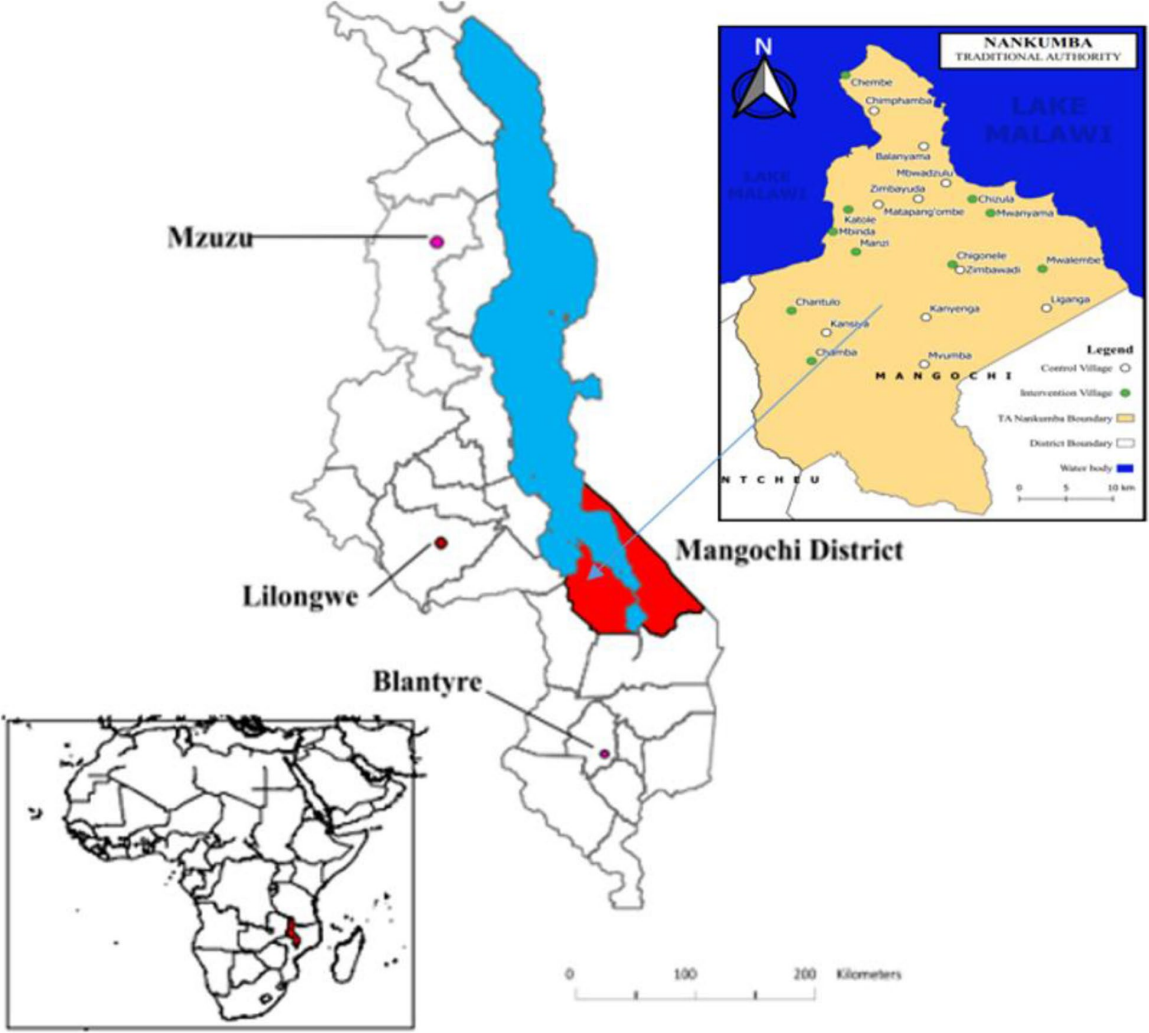
Map of Malawi showing Mangochi District (red), Lake Malawi blue), and Location of T.A. Nankumba (right side inset) with the study villages (intervention-green and control-white circles). Modified from source [21]

We conducted the RCT from January to December 2016 in the Namkumba area in Mangochi, one of the 12 districts in the southern region of Malawi. Mangochi district was prioritised for this intervention as it faces major challenges in maternal and child undernutrition, according to the Demographic Health Survey of 2010 [19]. Nankumba area is situated on the southern end of Lake Malawi (Fig. 1). We included pregnant women attending ANC from Monkebay, Namkhumba, Malembo and Nkope health centres. The population of the area is about 150,000 and the inhabitants' livelihood is mainly fishing or farming (depending on geographical location) [20].

### Selection and recruitment of study participants

All consenting pregnant women who were between their 9^th^ and 16^th^ gestational week, both primi- and multiparous, were eligible for inclusion. Moreover, they had to be available during the study period and had to intend to give birth at the health facilities within the study area. Women carrying multiple foetuses and those with severe illnesses were excluded from the study.

Twenty clusters, defined as a village which did not share common borders with other villages in the study, were eligible to be included and were mapped out in the study area. The villages were assigned STATA-generated random numbers to allocate them to either the intervention or the control group, generating ten clusters in each study group. The women from these clusters were identified by the study team in their communities and referred to the nearest designated local health centre where their pregnancy and the gestational age were confirmed and where they were also invited to consent to participate in the study.

### The intervention and data collection

The intervention addressed some of the underlying and contextual causes of undernutrition as per the UNICEF’s framework [22], such as poor dietary quality and factors usually resulting from inadequate: (i) knowledge of the benefits of good nutrition and nutrition dense foods, (ii) skills in food preparation that ensure the inclusion of diverse food groups in the diet, (iii) food processing that enhances absorption of nutrients from the gut, and (iv) motivation to put in the efforts needed to adhere to the recommended dietary guidelines. In the intervention, we therefore applied nutrition education, cooking demonstrations and home-based counselling. We used lay counsellors who were women from the same community (and who were already involved in community activities) to facilitate the education and counselling sessions. Moreover, we trained the lay counsellors in the intervention processes, and to use standard guidelines to ensure the implementation of the intervention.

The education component was offered before cooking demonstration to groups of women (6–20 women per group). Nutrition education was oriented towards enhancing individual understanding of the importance of eating diverse food, communicating the effects of diversified diet for mother- and child health, and conveying information about the food groups and their function in the human body. The cooking demonstrations were focused on teaching the women how to prepare foods that integrated the SFG dietary guidelines. These sessions were done once a month, during the week and utilised a whole afternoon. Prior to the session, the women were assigned to prepare the powder ingredients, process maize flour and mobilise all other resources needed. Most women attended about 4 sessions before they delivered.

We demonstrated on how to use the recipes that included ingredients made from food supplements turned into powder seasoning to make the meals more diversified. The powders were from anchovy (*Bonya*, dried small bony fish), groundnuts (*Nsinjiro*) and dried moringa tree leaves (*Chammwamba*). Anchovies are a good source of calcium, iron, and zinc, groundnuts are a good source of proteins and fats, while moringa leaves are good source of protein, vitamin B_2_, vitamin B_6_, vitamin C, iron, vitamin A and magnesium [23]. The recipes combined foods with high content of nutrients that were lacking in their usual diet. The most limiting foods in these settings were legumes while animal foods and vegetables were usually inaccessible at upland and lake shore household. *Nsima* was usually taken with one relish, which made the diet comprise of only two food groups of the required six. The most accessible main meal combination from upland was *Nsima* (a thick porridge), usually taken with only vegetables, whereas in the lakeshore areas *Nsima* was commonly taken with fish.

The diets in the communities participating in the study were predominantly plant-based and thus contained a high content of phytate and polyphenols that inhibit the absorption of iron, zinc and calcium [24]. The women thus got demonstrations on food processing that enables increased absorption of nutrients, such as soaking beans overnight before cooking and soaking whole grain maize for three days before turning it into maize meals used for making porridge and the staple food *Nsima*.

The practices promoted in this intervention were harmonised with local dietary patterns and food beliefs. Recipe development was guided by linear programming analyses of dietary intakes of pregnant women in the area. The project conducted Trials of Improved Practices to identify challenges with use of the recipes over a longer time which could hinder adoption of the recipes. In line with this, the recipes were tested among a comparable group of pregnant women in a non-study area.

We also offered home-based nutrition counselling to the women once a week to enhance intake according to the proposed dietary guidelines. Counselling sessions were offered weekly based on the individual needs and involved women with their families, aiming to promote their capabilities to source the required ingredients [25]. Each counsellor was expected to support up to twenty women within walking distance from the counsellor’s home. More information regarding delivery of the intervention has been previously described [20]. No intervention-related harm was detected.

### Measurements

A questionnaire that was partly adapted from a validated general nutrition knowledge questionnaire [26] was developed to collect sociodemographic data, nutrition knowledge and food consumption behaviours. Socioeconomic status was assessed using a household asset index based on eleven household items according to their monetary value and given scores very poor: ≤ 1.25; poor: 1.25–3.75; well-off: > 3.75) [27].

Women were included in the study between nine and sixteen weeks of gestation and the average exposure to the intervention was about 6 months. We assessed the study groups at inclusion (baseline) and at two weeks before expected delivery (study end-point) for differences in nutrition knowledge, perceptions and dietary habits. To assess nutrition knowledge, we asked the participants if they knew the SFG dietary guideline and if they could mention the food groups. Each food group mentioned weighted equally with the score of one, making a total score of six. We rated nutrition knowledge as low for scores 1–3, moderate for scores 4–5 and high for score 6 [28]. To assess perceptions, we asked the participants whether pregnant women have higher risk for malnutrition (yes or no). The women were also asked to evaluate their own dietary habits to determine if they were at risk for malnutrition (yes or no). We further assessed dietary habits through their recalled 24 h quantitative food consumption, from which we evaluated their scores using both the SFG and the DDS. Dietary diversification with use of SFG guidelines was applied using the same rating used for nutrition knowledge. The DDS for women (MDD-W) was based on 10 food groups as proposed by the Food and Agriculture Organisation [29]. The MDD-W is a dichotomous indicator based on 10 food groups and is considered the standard for measuring population-level dietary diversity in women of reproductive age [30, 31]. Each food group was weighted equally with the score of 1; hence the maximum possible score was 10. The MDD-W was rated as either poor (scores 0–4), moderate (scores 5–7) or high (scores ≥ 8). We also assessed the women’s self-reported nutrition behaviour using four questions, asking if they consumed diversified foods, healthy drinks and snacks, whole grains and skipped the main meals more frequently or not.

### Statistical analyses

For the RCT, we estimated the sample size based on the assumption that we would be able to detect a clinical meaningful mean difference in birth weight of 150 g between the control and intervention group [32]. Using a two-sided test, an alpha of 0.05 and a statistical power of 80%, we obtained a minimum sample size of 218 participants. In the current study we performed descriptive analyses using IBM SPSS Statistics 25 to assess the differences in baseline and study end-point characteristics between the two study groups, employing the Mann–Whitney U test for non-normal continuous variables and the Pearson chi-square test for associations between categorical variables. Further, the independent T-test was used to compare the mean nutrition knowledge score and dietary diversity score between the women in the two study groups.

We performed multilevel, multiple imputations of missing data using the function “mice” in the miceadds package in R We carried out 20 imputations under the assumption that the missing values were missing at random and exported the data to Stata. We then used three-level linear multilevel regression models with random intercept and random effect of time on participants at level 1 and village at level 2 to explore the association between nutrition knowledge and consumption of diversified diet. We adjusted the associations for maternal age, education, family size and food shortage. All statistical models were fitted using Stata SE 15 and the significance level was set at *p* < 0.05.

## Results

**Fig. 2:**
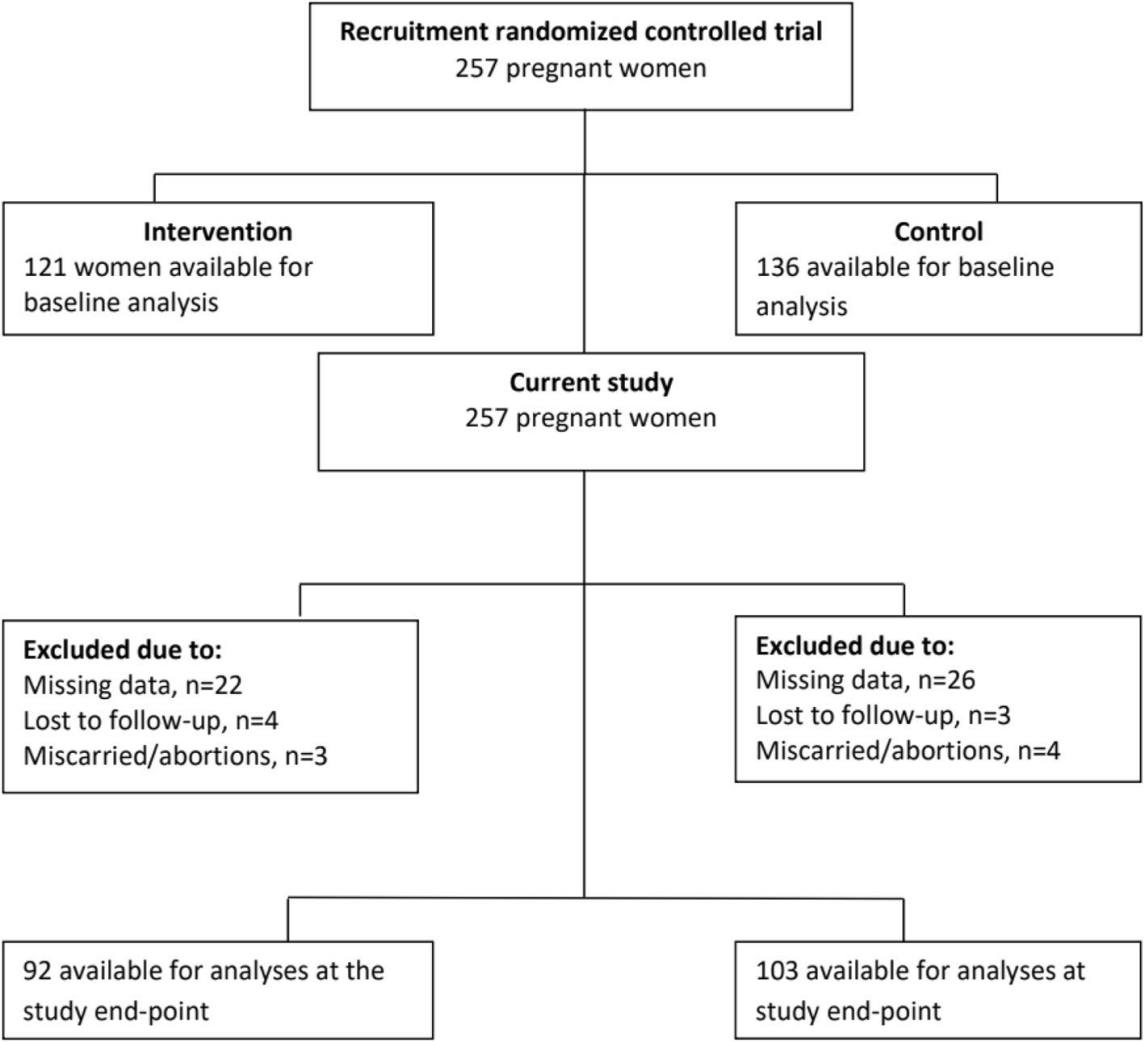
Flow chart showing the enrolment of participants into the two study groups

Of the 257 women who were recruited to the RCT, 195 (76%) were available for analysis at study end-point in the current study (Fig. 2). The study participants were mostly married, with 0 – 3 children, were either illiterate or had attended only primary school and were very poor (Table 1). Notably, the two study groups were well-balanced, indicating that the randomization process was adequate.

**Table 1:** Baseline characteristics of the study groups

Characteristic	Control (n = 103)	Intervention (n = 92)	*p*-value
Median (IQR) maternal age (years)	23 (18–28)	24 (19–30)	0.11
Median (IQR) number of previous births	1 (0–3)	2 (0–3)	0.12
Median (IQR) household size	5 (3–6)	5 (3–6)	0.06
Median (IQR) months of houshold food shortage last year	2 (1–5)	3 (1–6)	0.05
Geographic location (n, %)			
Lakeshore	60 (58.3)	43 (46.7)	0.11
Upland	43 (41.7)	49 (53.3)	
Socio-economic status (n, %)			
Very poor	29 (28.2)	31 (33.7)	0.56
Poor	43 (41.7)	39 (42.4)	
Well-off	31 (30.1)	22 (23.9)	
Maternal income (n, %)			
No income	83 (80.6)	75 (81.5)	0.87
Has income	20 (19.4)	17 (18.5)	
Marital status (n, %)			
Single	12 (16.5)	9 (4.3)	0.67
Married	91 (83.5)	83 (95.7)	
Education (n, %)			
Illiterate/primary	79 (78.6)	79 (83.7)	0.10
Secondary/above	24 (21.4)	13 (16.3)	
Household head gender (n, %)			
Female	9 (11.7)	7 (4.3)	0.77
Male	94 (88.3)	85 (95.7)	

*IQR* Interquartile range

As presented in Table 2, there were improvements in nutrition knowledge, perceptions and dietary habits in both study groups, but mostly in the intervention group. The results showed that the median nutrition knowledge scores using SFG rating among the women in the intervention and control group was 6 versus 3 at study end-point. Significantly more women in the intervention group (58.4%) compared to the controls (23.6%) attained the highest score (6) for nutrition knowledge at study-end point. Most (> 80%) women from both study groups expressed that pregnant women in general are at risk of malnutrition. However, most of the study women did not consider themselves as being at risk of malnutrition. Table 2 shows that at study-end point median SFG score was 5 for both study groups, whereas median MDD-W was 7 and 6 in the intervention group and controls, respectively. Although the differences between the two study groups were small at study end-point, they reached significance. Regarding nutritional behaviour, we found that at study end-point, the majority of women in the intervention group to a larger extent than the controls, expressed that they frequently consumed a diversified diet, healthy snacks and whole grains.

**Table 2:** Nutrition knowledge, perceptions and dietary intake at baseline and at study end-point

**Outcomes**	**Baseline**			**Study end-point**		
	**Control (n = 78)**	**Intervention (n = 68)**	***p*-value**	**Control (n = 78)**	**Intervention (n = 68)**	***p*-value**^**a**^
**Knowledge of SFG (% yes)**						
Staple	23.3	25.0	0.78	38.3	61.7	< 0.01
Animal Food	23.3	32.6	0.14	37.1	62.9	< 0.01
Vegetables	29.1	41.3	0.08	40.5	59.5	< 0.01
Legumes	21.4	26.7	0.43	38.1	61.9	< 0.01
Fruits	21.4	27.5	0.32	38.6	61.4	< 0.01
Fats	18.4	23.9	0.35	43.0	66.0	< 0.01
**SFG knowledge scores**						
Mean (IQR) nutrition knowledge scores	0 (0–2)	0 (0–4)	0.52	3 (0–5)	6 (3–6)	< 0.01
Low scores (%)	82.5	73.6	0.28	62.1	26.4	< 0.01
Moderate scores (%)	5.8	11.0		14.6	15.4	
High score (%)	11.7	15.4		23.6	58.4	
**Nutrition perceptions (%)**						
Know that pregnant women have high risk for malnutrition	80.6	80.4	0.87	81.6	91.3	0.05
Perceptions of not being at-risk for malnutrition	26.2	28.3	0.75	23.2	9.8	< 0.01
**Median (IQR) dietary diversity scores**						
SFG	4 (3–5)	4 (3–6)	0.35	5 (4–5)	5 (4–6)	< 0.01
MDD-W score	5 (4–5)	5 (4–7)	0.19	6 (5–6)	7 (5–8)	< 0.01
**Nutrition behaviour (% yes)**						
Consumption of diversified foods frequently	26.2	25.0	.87	43.7	62.6	< 0.01
Missing main meals less frequently	29.1	28.3	0.89	55.3	78.3	< 0.01
Taking healthy drinks and snacks frequently	15.8	19.1	0.55	24.5	42.9	< 0.01
Eating whole grain frequently	21.4	22.8	0.81	65.7	90.1	< 0.01

^a^The *p*-values relate to the differences between the two study groups The n varies due to missing/incomplete data

*IQR* Interquartile range, *SFG* Six Food Group, *MDD-W* Minimum dietary diversity for woman

**Fig. 3:**
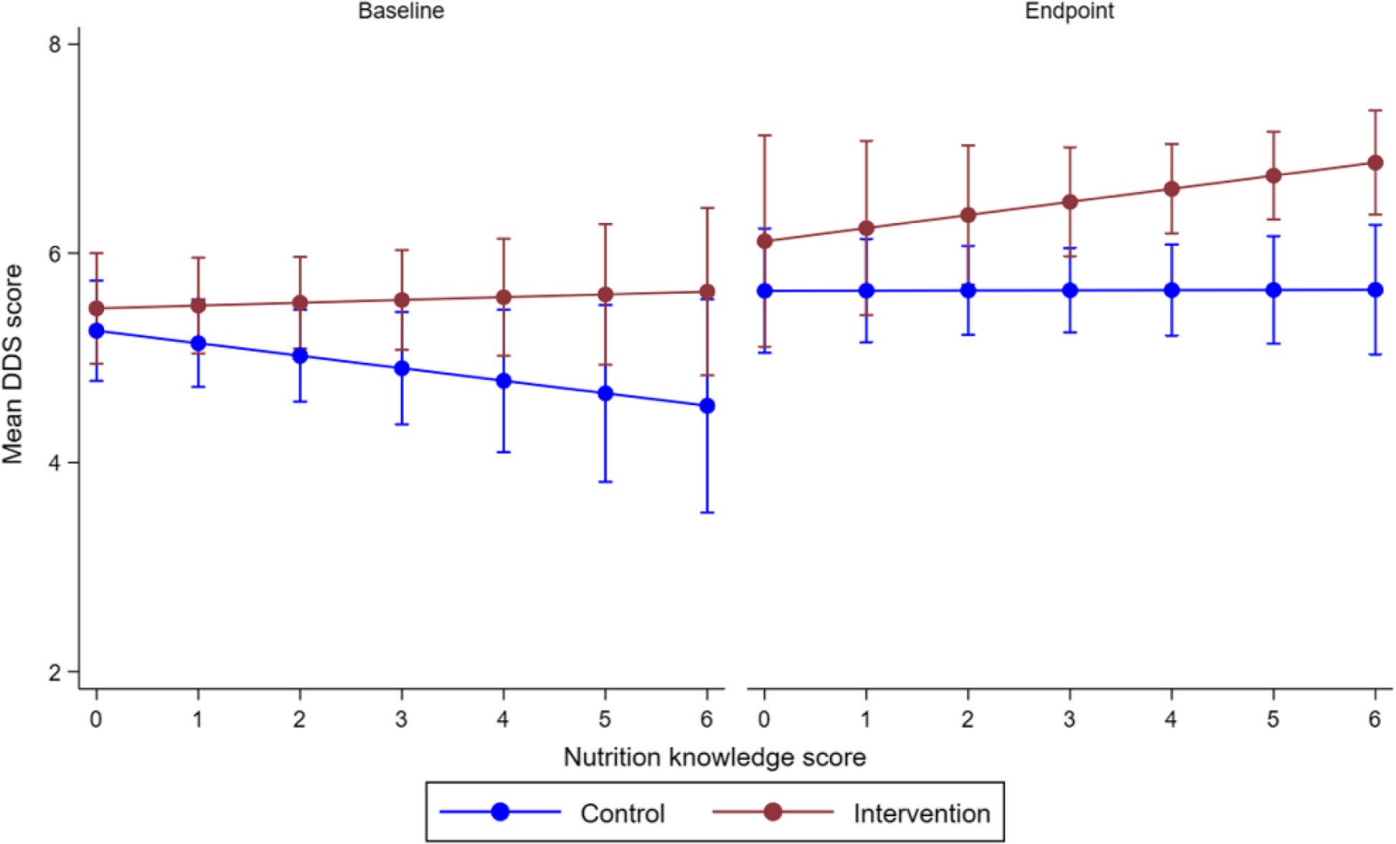
Association between nutrition knowledge and dietary diversity score. The values represent marginal means with 95% confidence intervals, and adjusted for age, education and household size

Figure 3 shows that an increase in nutritional knowledge between baseline and end-point improved MDD-W in the intervention group, but not among the controls. Moreover, a comparison of the two study groups also showed that improvements in nutritional scores ≥ 4 significantly increased MDD-W in the intervention than in the control. However, such improvements in MDD-W between the two study groups were not observed at baseline.

**Fig. 4:**
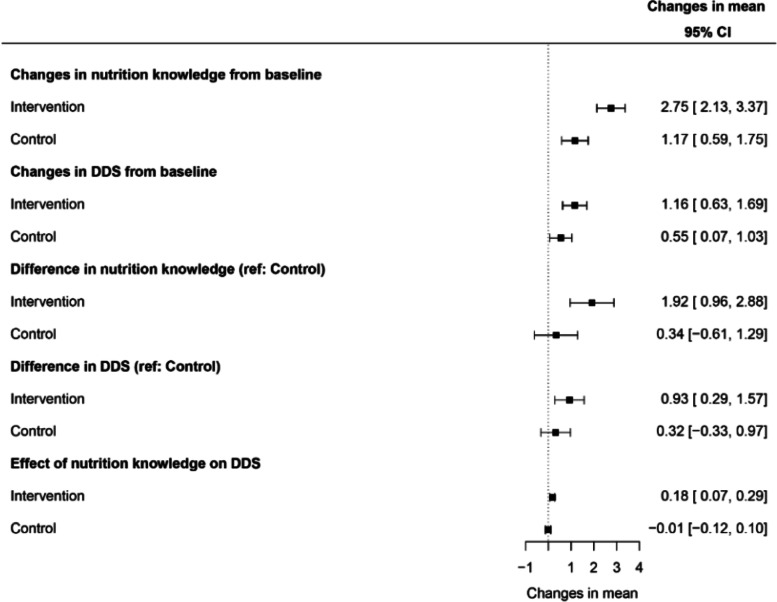
Mean changes in nutrition knowledge and diet diversity score within and between the two study groups. Values are mean (95% confidence intervals) changes in nutrition knowledge and DDS. Estimates to the right of the vertical line (dotted) in the forest plot represents increases in scores whereas estimates to the left represent decreases of the scores. The 95% confidence intervals cutting through the vertical line represents results that are not statistically significant. CI, confidence interval; DDS, diet diversity score

Figure 4 portrays mean changes in nutrition knowledge and MDD-W within and among the two study groups. There was a significant improvement in both nutrition knowledge and MDD-W in the intervention compared with the control group. We also found that nutrition knowledge significantly improved MDD-W in the intervention group, but not among the controls.

## Discussion

This study compared the effects between nutrition education and counselling offered through routine ANC (control group) with a supplementary community-based nutrition education and counselling (intervention group) on nutrition knowledge and dietary behaviour of pregnant women in rural Malawi. Our results showed that both nutrition knowledge and dietary diversity improved in both study groups, but the improvements were significantly higher in the intervention group at study end-point. Increase in nutrition knowledge was associated with improved dietary diversity only among women in the intervention group. Moreover, women in the intervention group significantly improved their nutrition perceptions and behaviour.

Utilisation of ANC among pregnant women in rural Malawi is at 80% [2], and of these nearly 90% receive nutrition counselling as part of this care according to the Malawian Demographic Health Survey (2015–2016) [33]. The nutrition education offered is based on the National Nutrition Guidelines [33], which note that during pregnancy, women should eat meals containing ingredients from each of the SFG and eat two extra meals per day. The SFG dietary guideline is easy to apply in a low-resource settings and its rating corresponds to the MDD-W rating in terms of dietary adequacy [20].

Nutrition education and counselling are expected to improve pregnant women’s food literacy, which corresponds to an advanced level of nutrition knowledge and understanding [34]. Our results show that exposure to the intervention enabled the pregnant women to enhance their food literacy as demonstrated by the fact that more than half obtained higher scores in nutrition knowledge and a diversified diet. Moreover, those in the intervention group who scored high in nutrition knowledge had a significantly higher MDD-W compared to the controls at study end-point. These findings demonstrate that an adequate degree of nutrition literacy is required to enhance the responsiveness to health advice [7]. Nutrition literacy in this context was demonstrated through the women’s ability to understand and interpret information regarding the SFG dietary guidelines whereas the transition to a more diversified diet represented sufficient competence to make appropriate nutrition decisions [7]. We may then deduce that the intervention was effective in enhancing food literacy which is exhibited through a combination of competencies in nutrition literacy and the ability to apply that information to make appropriate nutrition decisions. These results are consistent with previous research and theories that have identified success of nutrition interventions having focus on a combination of nutrition awareness, motivation and environmental support for action [5]. The improvements observed among the women were thus a product of different components in a series of interventions beyond a mere provision of nutrition education, as is the case for ANC services.

Our intervention targeted groups, households and individuals with motivation and social support for the pregnant woman to adhere to the recommended dietary habits. This approach was in line with the evidence that interventions are more effective when they aim at enhancing the actual behaviour or actions being important for behaviour change rather than on impartation of knowledge only [5, 35].

We attribute the nutrition knowledge gaps among the controls to poor quality of nutrition education and counselling. We observed that it might not be possible to provide a comprehensive nutrition program within the ANC setting due to time management constraints. The education component of ANC services is offered as group education sessions that last maximum 20 min and do not include individualised targeted interventions. A systematic review on processes and outcomes of antenatal care found that ANC education sessions were conducted hurriedly and in a less supportive way due to lack of time and inadequate skills [35]. These circumstances may have yielded limited nutrition awareness and thus unlikely for the women to remember or retain the information [36]. Other studies from sub-Saharan Africa also postulate that the poor quality of ANC is a result of low skills among health workers responsible for the nutrition education [11, 37]. Apparently, health workers often do not utilise the standard guidelines, leading to variations in content and approaches [34]. Reportedly, there was no difference in nutrition knowledge between pregnant women attending ANC and those who did not [11]. Moreover, residing in low-resource settings women often under-utilise ANC so that those who make fewer antenatal visits are more likely to miss nutrition counselling [2, 37]. Although ANC services remain the most accessible source for nutrition education, they may not permit the roll-out of a comprehensive nutrition education. Under such circumstances, pregnant women should be encouraged to access supplementary nutrition programs, especially those aired in media, e.g. on radio or television. In support of this, one Ethiopian study showed that this approach proved effective in improving nutrition habits among pregnant women [38].

Most of the women (over 50%) in both study groups attained a diversified diet corresponding to a rating of “medium” according to DDS, i.e. more than five food groups. These results show a better improvement in consumption of diversified diet when compared with findings from a recent study from rural Malawi in which only 31% of participants ate more than five food groups [2]. We found a positive association between improved nutrition knowledge and dietary diversification only among women in the intervention group. Similar observation was made in a study from Ethiopia [39]. These authors concluded that in-depth nutrition awareness enabled pregnant women to become more concerned of the effects of malnutrition on maternal and child health, and thus drive them into action to avoid that. Adequate nutrition knowledge may also explain the association between socioeconomic status and dietary quality as it may enhance the ability to identify various foods with the same nutritive components which may offer a varied and heathier food purchasing behaviour [40]. Furthermore, this may limit the tendency of wasting money on expensive foods when cheaper alternatives with the same nutrition value are available in the local market.

The main strength of our study is the randomized design adopting pragmatic measuring tools that can produce robust data in a challenging low-resource setting as rural Malawi. Although the intervention consisted of a comprehensive community-based nutrition education targeting several factors known to influence dietary habits, other factors that may influence such habits such as taste, convenience, social norms, religious and cultural beliefs, were not addressed [41, 42].

## Conclusion

Our study provides evidence that poor nutrition knowledge is a barrier to consumption of a healthy diet during pregnancy, a period where women residing in low-resource settings are at increased risk of malnutrition. Thus our results emphasise the relevance for augmenting nutrition education offered at ANC with supplementary and community-based nutrition interventions. In addition, we suggest that efforts should be made to strengthen the education component of ANC services as these are decentralised and locally accessible in most low- and middle-income countries and may therefore contribute to higher nutrition literacy regarding a healthy diet during pregnancy.

## Data Availability

The datasets used and analyzed during the current study are available from the corresponding author on reasonable request.

## Abbreviations

ANC: Antenatal care
DDS: Diet diversity score
LBW: Low birth weight
MDD-W: Minimum dietary diversity for women
RCT: Randomized controlled trial
SFG: Six food group
